# The third model of Bax/Bak activation: a Bcl-2 family feud finally resolved?

**DOI:** 10.12688/f1000research.25607.1

**Published:** 2020-08-06

**Authors:** Xu Luo, Katelyn L. O'Neill, Kai Huang

**Affiliations:** 1 Eppley Institute for Research in Cancer and Allied Diseases, Fred & Pamela Buffett Cancer Center, University of Alaska Medical Center, Omaha, ME, 68198-7696, USA; 2 Department of Pathology and Microbiology, University of Nebraska Medical Center, Omaha, NE, 68198-6805, USA

**Keywords:** Apoptosis, Bcl-2 family, Bax, Bak, BH3-only proteins, Bid, Bim, Bad, Bcl-xL, Mcl-1, Activator, Sensitizer, Direct activation, Indirect activation, Membrane-mediated Spontaneous activation, Mitochondrial outer membrane, auto-activation, de novo activation, retro-translocation, BH3 mimetics

## Abstract

Bax and Bak, two functionally similar, pro-apoptotic proteins of the Bcl-2 family, are known as the gateway to apoptosis because of their requisite roles as effectors of mitochondrial outer membrane permeabilization (MOMP), a major step during mitochondria-dependent apoptosis. The mechanism of how cells turn Bax/Bak from inert molecules into fully active and lethal effectors had long been the focal point of a major debate centered around two competing, but not mutually exclusive, models: direct activation and indirect activation. After intensive research efforts for over two decades, it is now widely accepted that to initiate apoptosis, some of the BH3-only proteins, a subclass of the Bcl-2 family, directly engage Bax/Bak to trigger their conformational transformation and activation. However, a series of recent discoveries, using previously unavailable CRISPR-engineered cell systems, challenge the basic premise that undergirds the consensus and provide evidence for a novel and surprisingly simple model of Bax/Bak activation: the membrane (lipids)-mediated spontaneous model. This review will discuss the evidence, rationale, significance, and implications of this new model.

## Introduction

Apoptosis is a highly regulated form of cell death playing important roles in embryonic development, tissue homeostasis, and disease progression ^1^. In response to various death stimuli, cells often initiate a mitochondria-dependent apoptotic pathway that involves mitochondrial outer membrane permeabilization (MOMP), formation of the apoptosome, and the activation of effector caspases ^2^. Generally considered the “point of no return”, MOMP irreversibly commits cells to apoptosis through the actions of Bax and Bak, two functionally similar effector proteins of the Bcl-2 family ^3^.

**Figure 1.**
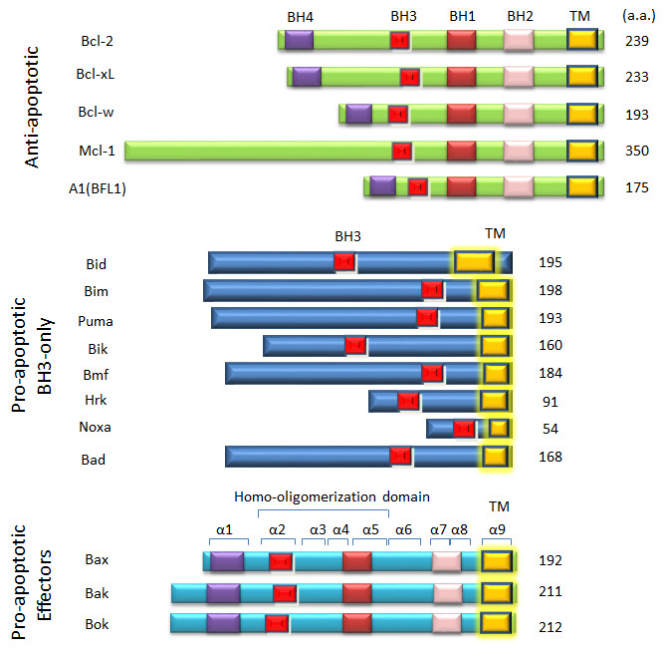
Schematic of Bcl-2 family proteins. Bcl-2 family proteins (human) are classified into three groups: the pro-apoptotic BH3-only proteins, the pro-apoptotic effector proteins Bax, Bak, and Bok, and the anti-apoptotic Bcl-2 proteins. Each family member contains at least one of the four Bcl-2 homology (BH) domains, BH1–4. Bax has nine α-helices, with helix 9 serving as a mitochondrial-targeting sequence ^13^. Helices 2–5 constitute the homo-oligomerization domain of Bax ^18, 42, 52^. a.a., amino acids; TM, transmembrane.

Since the identification of Bcl-2 in human follicular lymphoma ^4, 5^ and its discovery as the first anti-apoptotic oncogene ^6^ over three decades ago, 17–18 proteins that share at least one of the four Bcl-2 homology domains (BH1–4) have been identified in the mammalian system as Bcl-2 family proteins ^7^, with the last member, Bmf, identified in 2001 ^8^. The Bcl-2 family proteins are classified into anti-apoptotic (Bcl-2, Bcl-xL, Bcl-w, Mcl-1, and A1), pro-apoptotic effector (Bax, Bak, and Bok), and pro-apoptotic BH3-only proteins (Bad, Bid, Bik, Bim, Bmf, Hrk, Noxa, and Puma) ^9^ ( Figure 1). Three types of interactions—1) BH3-only–anti-apoptotic, 2) anti-apoptotic–Bax/Bak, and 3) BH3-only–Bax/Bak—appear to govern the functional relationships among the Bcl-2 family members during apoptosis ^10, 11^.

As the requisite effectors of MOMP, both Bax and Bak are kept in check under normal conditions. Bax is primarily in the cytosol as a globular-shaped monomeric protein consisting of nine α-helices, with helix 9 fitting into a hydrophobic groove ^12– 14^. Bak has a similar structure ^15^ but is localized to the mitochondrial outer membrane (MOM) through its helix 9. Both Bax and Bak are activated during apoptosis ^16^. The active forms of Bax/Bak are known to homo-oligomerize in the MOM, forming proteinaceous/lipidic pores that are responsible for the release of cytochrome c and other apoptogenic factors ^17, 18^. The BH3-only and the anti-apoptotic Bcl-2 proteins regulate MOMP by promoting or suppressing the activation of Bax/Bak, respectively ^9, 11, 19^.

Bok has recently been shown to complement Bax/Bak in developmental cell death ^20^ and to function as a non-canonical effector of MOMP in response to proteasome inhibition ^21^. However, since the loss of both Bax and Bak is sufficient to block apoptosis induced by most apoptotic stimuli ^22^, the role of Bok in apoptosis remains unclear.

As both Bax and Bak undergo a dramatic transformation from benign proteins to lethal effectors during apoptosis, the mechanism of their activation has been the subject of intense debate and is considered to be the “holy grail” of apoptosis research ^16, 23^. Two competing but not mutually exclusive models, direct and indirect activation, had previously been proposed.

## Direct activation

**Figure 2.**
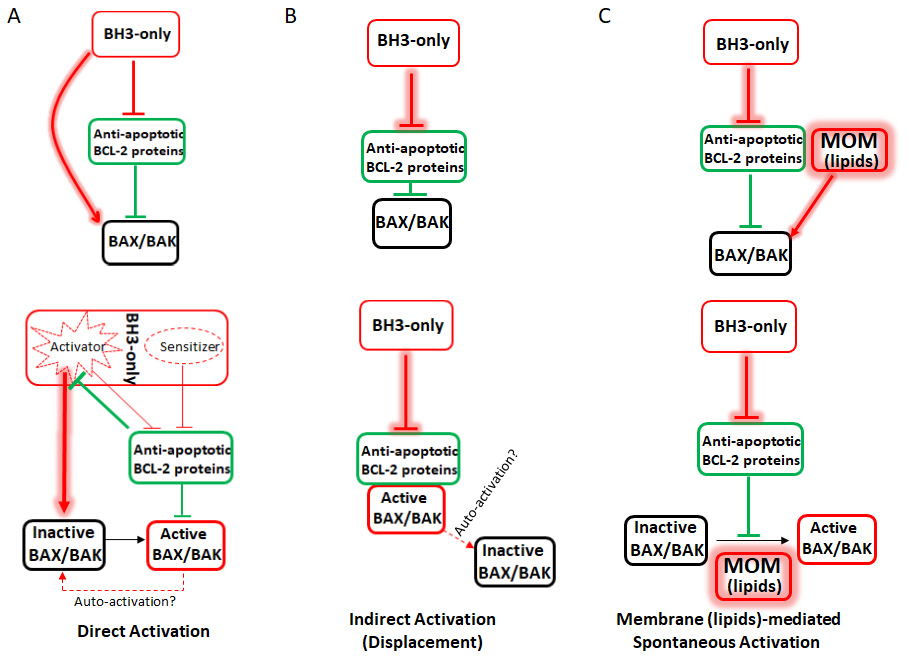
Comparison of the three models of BH3-only mediated Bax/Bak activation. **A**) The direct activation model classifies BH3-only proteins as either activators (i.e. Bid, Bim, and Puma) or sensitizers (Bad). When not sequestered by anti-apoptotic Bcl-2 proteins, the activators directly bind to and activate Bax/Bak. Sensitizers cannot directly activate Bax/Bak but can bind to anti-apoptotic Bcl-2 proteins. To initiate apoptosis, the sensitizers bind to the anti-apoptotic Bcl-2 proteins, which release the activators from sequestration and allow them to directly activate Bax/Bak. **B**) The indirect activation model hypothesizes that Bax/Bak are constitutively active but are sequestered by the anti-apoptotic Bcl-2 proteins. During apoptosis, the BH3-only proteins bind to the anti-apoptotic Bcl-2 proteins and displace active Bax/Bak, which then damage the mitochondria. It is also hypothesized that active Bax can recruit inactive cytosolic Bax to the mitochondria through auto-activation. **C**) The membrane (lipids)-mediated spontaneous activation model hypothesizes that during apoptosis, the BH3-only proteins bind to and neutralize the anti-apoptotic Bcl-2 proteins. This allows the mitochondrial outer membrane (MOM) lipids to serve as the direct activator of Bax/Bak, as Bax freely migrates to the MOM through free diffusion. Helix 9 mediates membrane association for both Bax/Bak, and such association results in spontaneous activation of Bax/Bak in the lipid bilayer of the MOM. Similar to the indirect activation model, the BH3-only proteins’ only function is to inactivate the anti-apoptotic Bcl-2 proteins. However, in the membrane (lipids)-mediated spontaneous (MMS) model, both Bax and Bak are directly activated by the MOM. Figure 2c is adapted from Figure 5b of our previous paper in Cell Research ^59^.

The first report of the direct interaction between a BH3-only protein and Bax/Bak was the identification of Bid as a BH3-containing death ligand that binds Bax. This finding led to the hypothesis that BH3-only proteins bind and activate Bax/Bak to initiate apoptosis ^24^. Subsequently, it was found that some BH3 domains, i.e. BidBH3 or BimBH3, have the ability to directly activate Bax/Bak and cause the release of cytochrome c from purified mitochondria. On the other hand, BH3 peptide from Bad (BadBH3) did not directly activate Bax/Bak but was able to bind to Bcl-2, displace BidBH3, and sensitize mitochondria to BidBH3-induced cytochrome c release ^25^. These findings formed the basis for the “direct activation” model, in which the BH3-only proteins are classified into two groups: the activators, which can directly activate Bax/Bak but can also be sequestered by the anti-apoptotic Bcl-2 proteins, and the sensitizers, which cannot directly activate Bax/Bak but are able to bind to the anti-apoptotic Bcl-2 proteins and sensitize cells to apoptosis. To induce apoptosis, the sensitizers bind the anti-apoptotic Bcl-2 proteins and displace the activators, which in turn directly bind to Bax/Bak, allosterically triggering their activation ^25– 28^ ( Figure 2A).

Although the initial classification listed Bid and Bim as direct activators because of the ability of the BH3 domain or the full-length protein to release dyes of large molecular weight from liposomes in the presence of inactive Bax ^25, 28– 32^, the list of direct activators has grown over the years to include Puma, Noxa, Bik, Bmf, and Hrk, although a range of potency has been reported ^26, 33– 39^. On the other hand, Bad has been a classic sensitizer, as the BadBH3 peptide has been used as a negative control for direct activators in liposome assays ^25, 28^.

The interaction between a direct activator and Bax/Bak has been difficult to detect. This led to the hypothesis that activators only transiently engage Bax/Bak and activate them through a “hit and run” mechanism. The binding interface between an activator and Bax/Bak, while of great interest, has been controversial. NMR analysis provided evidence that the BH3 peptide from Bim transiently binds to a “trigger site” between helix 1 and helix 6 ^40, 41^. However, other studies using Bax/Bak mutants that lack helix 9 found that a BidBH3 peptide binds the hydrophobic groove ^42– 45^. The discrepancy was largely resolved by new studies suggesting that the initial binding is at the trigger site and that the ensuing conformational changes allow a more stable interaction between the BH3 peptide and the hydrophobic groove ^46, 47^. However, the biological significance of binding at the trigger site has recently been challenged ^48, 49^.

Over the years, the direct activation model has evolved into several variants. The embedded together model emphasizes the importance of the membrane as a platform for all of the interactions among the Bcl-2 family proteins during Bax/Bak activation ^50, 51^, the unified model emphasizes that the anti-apoptotic Bcl-2 proteins not only sequester the activator BH3-only proteins but also suppress or sequester the activated Bax/Bak ^30^, and the interconnected model is highly analogous to the unified model but also contains a feed-forward mechanism of auto-activation ^36^. One unifying feature of these models is the direct attack and engagement of Bax/Bak by the activator BH3-only proteins as the initial triggering event leading to Bax/Bak activation.

Since the beginning of the last decade, direct activation has been widely accepted as the mechanism of Bax/Bak activation during apoptosis ^10, 53, 54^. However, as much of the supporting evidence has come from *in vitro* studies ^25, 28, 31, 32, 41– 43^, the physiological significance of this model has not been fully addressed.

## Indirect activation

Indirect activation, or the “displacement” model, was first proposed in two important studies. First, Bak was shown to be normally sequestered by Bcl-xL and Mcl-1 but displaced by BH3-only proteins ^55^. Second, in direct opposition to the direct activation model, Willis *et al*. demonstrated that apoptosis can be induced by “sensitizer” BH3-only proteins or mutants of “activators” that are unable to bind Bax/Bak, even in cells that are deficient for the three activator BH3-only proteins Bid, Bim, and Puma ^56^. These results gave rise to the indirect activation model, which proposed that the effectors Bax/Bak are constitutively active but are normally sequestered by the anti-apoptotic Bcl-2 proteins. During apoptosis, BH3-only proteins bind to anti-apoptotic Bcl-2 proteins and indirectly activate Bax/Bak by displacing and releasing sequestered active Bax/Bak ^57^ ( Figure 2B, top).

Although this model is consistent with the activation of Bak, it has difficulty explaining the activation of Bax. It was therefore hypothesized that a small fraction of Bax is constitutively active but is sequestered by the anti-apoptotic Bcl-2 proteins on the mitochondria. The BH3-only proteins release the active Bax, which in turn recruits cytosolic Bax to the mitochondria and activates it through an auto-activation mechanism ^58^ ( Figure 2B, bottom). However, this hypothesis does not address the origin of the fraction of active Bax and the physiological relevance of auto-activation. Furthermore, as the indirect activation model was based on the key observation of Bax/Bak activation in Bid/Bim/Puma-deficient cells, the existence of additional activator BH3-only proteins made it more difficult to fully dissociate from the direct activation model ^26, 33– 36^. It is worth mentioning that in an elegant genetic knockin study in mice, Merino *et al*. compared the pro-apoptotic activities of wild-type Bim and its BH3-replacement mutants and presented data to suggest that neutralization of the anti-apoptotic Bcl-2 proteins is required but not sufficient for maximal apoptosis, suggesting the presence of both indirect and direct activation *in vivo* ^60^.

## Functional redundancy and functional/physical connections: the Achilles’ heel of Bcl-2 research

A salient feature of the Bcl-2 family proteins is functional redundancy due to the presence of eight BH3-only, five anti-apoptotic, and two effector proteins. With multiple proteins sharing similar targets and functions in the same cell, it is difficult to pinpoint the real target, function, and mechanism of action of a particular protein ^23, 61, 62^.

As Bax and Bak are functionally redundant in many situations, our understanding of their functions was severely limited until a seminal study in which mouse embryonic fibroblasts (MEFs) doubly deficient for Bax and Bak were shown to be highly resistant to multiple apoptotic stimuli ^22^. This breakthrough ended years of frustration and established Bax and Bak as functionally equivalent and critical effectors of MOMP. However, the investigation into the functions and mechanisms of BH3-only proteins had been more challenging owing to the presence of eight BH3-only proteins, with seven of them potentially functioning as direct activators ^33– 36^. In efforts to differentiate direct and indirect activation, loss-of-function studies have been carried out to eliminate the direct activators by generating Bid/Bim double knockout (DKO), Bid/Bim/Puma triple knockout (TKO), Bid/Bim/Puma/p53 quadruple knockout (QKO), and Bid/Bim/Puma/Noxa QKO cells ^36, 56, 63– 65^. However, these cell models are insufficient to address this critical issue because of the presence of additional direct activators (i.e. Bik, Hrk, and Bmf) in those cells ^33– 35^.

The second challenge is the complex functional and physical connections among the Bcl-2 family proteins. The anti-apoptotic Bcl-2 proteins interact with at least some of the BH3-only proteins constitutively. Therefore, in cells deficient for certain BH3-only proteins—notably, the Bid/Bim/Puma TKO and Bid/Bim/Puma/Noxa QKO cells ^36, 63^—the loss of the BH3-only proteins should effectively increase the concentration of “unsequestered” or “active” Bcl-xL, Bcl-2, and Mcl-1, etc., whose total protein levels may not change. This increase would mean that these cells should have a greater capacity to sequester or suppress the active Bax/Bak and should at least in part explain the resistance of these TKOs and QKOs to apoptosis stimulation or the expression of the sensitizer protein Bad. Because of such complexity, vastly different conclusions have been drawn to either support or disprove the role of the activator proteins and the distinction between activators and sensitizers ^26, 36, 59, 66^.

## “OctaKO” and “Bcl-2 allKO” cells: essential tools to reveal that direct activation is not required for BH3-only-mediated Bax/Bak activation and to establish the membrane (lipid)-mediated spontaneous model

In the past decade, the list of direct activator BH3-only proteins has grown to include Bid, Bim, Puma, Hrk, Noxa, Bik, and Bmf, which bind both Bax/Bak and the anti-apoptotic Bcl-2 proteins. Such a functional redundancy makes it near impossible to separate the functions of the direct activators and the sensitizers unless all of the direct activators are eliminated ^61^. The advent of CRISPR technology made it possible to remove this roadblock.

Through a sequential use of the CRISPR/Cas9 technology, HCT116 cells that lack all eight BH3-only proteins were generated and named OctaKO cells ^66^. This allowed for the interrogation of the function of individual BH3-only proteins without the participation of other BH3-only proteins. In this cell model, elimination of the two anti-apoptotic Bcl-2 proteins Bcl-xL and Mcl-1 by siRNA, pharmacological inhibition, or genetic elimination induced Bax/Bak activation and apoptosis efficiently. These results, for the first time, demonstrated that none of the direct activator BH3-only proteins is necessary for Bax/Bak activation once Bcl-xL/Mcl-1 are neutralized.

Similar strategies were used to generate the Bcl-2 allKO cells, which lost all eight pro-apoptotic BH3-only proteins, Bnip3 and Nix (non-apoptotic BH3-only proteins), the anti-apoptotic Bcl-2 proteins (Bcl-2, Bcl-xL, Mcl-1, A1, and Bcl-w), and Bax/Bak ^66^. These cells for the first time provided a clean slate to examine the functional relationship, through reconstitution, among Bcl-2 family proteins. Surprisingly, even minute amounts of Bax or Bak, re-introduced through a Dox-inducible expression system, were sufficient to kill these cells, and such killing was easily blocked by Bcl-xL. The highly efficient activation of Bax/Bak in Bcl-2 allKO cells is in stark contrast with the prediction from the direct activation model, which suggests that Bax should stay in the cytosol in the absence of an activator BH3-only protein. Importantly, mutants of Bax and Bak that lack their respective helix 9 (transmembrane [TM] domain) are incapable of homo-oligomerization and killing, and yet such activity was completely restored by the attachment of a heterologous TM domain, strongly suggesting that MOM association is sufficient for Bax/Bak activation ^66^.

**Figure 3.**
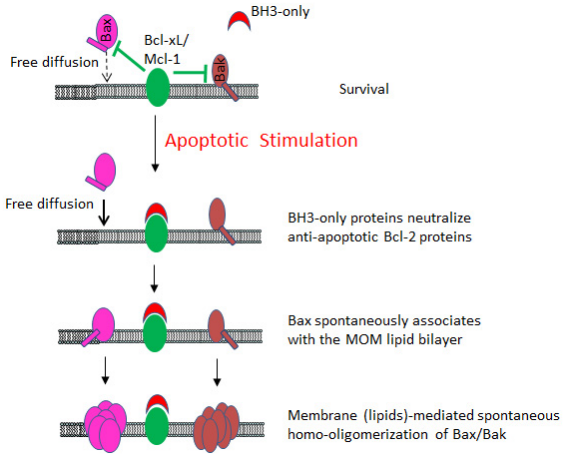
Membrane (lipids)-mediated spontaneous activation of Bax/Bak by the BH3-only proteins. In this model, upon apoptotic stimulation, the BH3-only proteins neutralize/inactivate the anti-apoptotic Bcl-2 proteins Bcl-xL/Mcl-1. Such inactivation allows Bax to freely migrate to the mitochondrial outer membrane (MOM) through the interaction between helix 9 and the lipid bilayer. Anchored to the MOM through helix 9, both Bax and Bak spontaneously homo-oligomerize and form apoptotic pores within the lipid millieu. This figure is adapted from Figure 7 of our previous paper in Genes & Development ^66^.

These results formed the basis for a novel model of Bax/Bak activation, the membrane (lipid)-mediated spontaneous model (MMS), in which the BH3-only proteins activate Bax/Bak by inactivation or neutralization of the anti-apoptotic Bcl-2 proteins, instead of direct activation. This allows the Bax/Bak molecules to freely migrate to and diffuse in the MOM, homo-oligomerize, and form pores ( Figure 2C, Figure 3). In this model, the driving force of Bax/Bak activation is essentially free diffusion and the well-documented MOM association through helix 9 ^66^. Consistent with this model, cytosolic Bax was reported to be constitutively targeted to the mitochondria in non-apoptotic cells ^67, 68^.

## What are the targets of BH3-only proteins: anti-apoptotic Bcl-2 proteins, Bax/Bak, or both?

**Figure 4.**
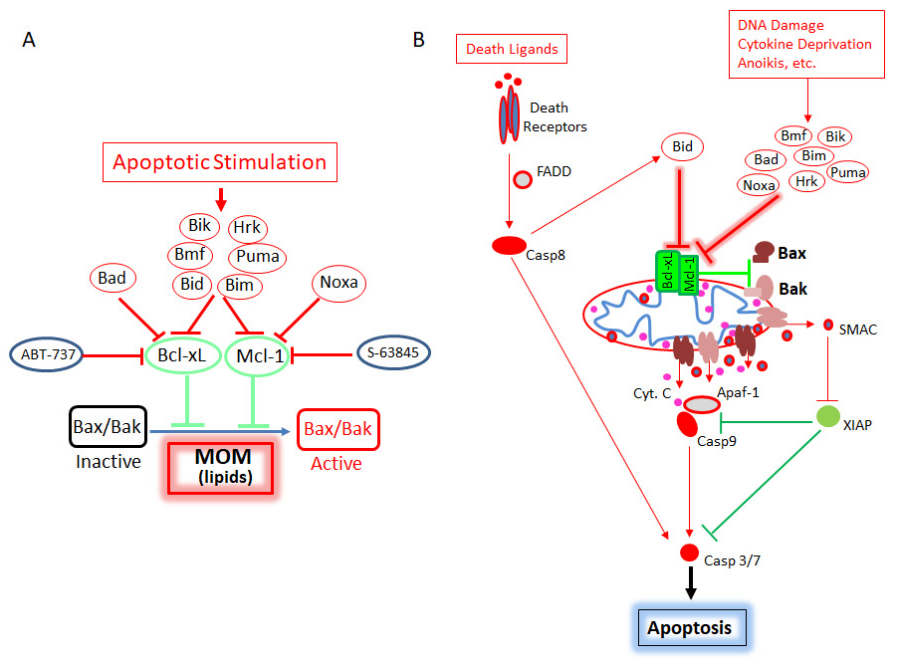
Apoptotic signaling through membrane (lipids)-mediated spontaneous activation of Bax/Bak **A**) The intrinsic (mitochondrial-dependent) apoptotic pathway based on the membrane (lipids)-mediated spontaneous (MMS) Bax/Bak activation model. **B**) The intrinsic (mitochondrial-dependent) and extrinsic (death receptor-mediated) apoptotic pathways based on the MMS activation model. Apaf-1, apoptotic protease activating factor-1; Casp, caspase; Cyt c, cytochrome c; FADD, Fas associated death domain protein; MOM, mitochondrial outer membrane; SMAC, second mitochondria-derived activator of caspase; XIAP, X-linked inhibitor of apoptosis protein.

Our understanding of the functions of the BH3-only proteins was significantly enhanced when Chen *et al*. defined the binding specificity of various BH3-only proteins toward different anti-apoptotic Bcl-2 proteins ^69^. Using peptides of the BH3 domains of the BH3-only proteins, Chen *et al*. found that while Bid, Puma, and Bim are promiscuous in binding all five anti-apoptotic Bcl-2 proteins, Bad only selectively binds to Bcl-w, Bcl-xL, and Bcl-2, and Noxa binds only Mcl-1 and A1. The combination of Bad and Noxa potently induced apoptosis ^69^. Using derivatives of the OctaKO cells that also lack Bcl-xL or Mcl-1, the Huang *et al*. study confirmed and refined these findings by demonstrating that while Bad and Noxa preferentially bind Bcl-xL and Mcl-1, respectively, tBid, Bim, Puma, Bmf, Bik, and Hrk are promiscuous binders toward both ^59^ ( Figure 4A).

The critical role of Bcl-xL and Mcl-1 in cell survival was first demonstrated in HeLa cells in which siRNA knockdown of both Bcl-xL and Mcl-1 resulted in robust apoptosis ^70, 71^. This assay was subsequently carried out in multiple cell lines with deficiencies in several BH3-only proteins and ultimately the OctaKO cells. Without exception, the double siRNA knockdown induced robust apoptosis, strongly suggesting that Bcl-xL and Mcl-1 are the major targets of the BH3-only proteins during apoptosis ^36, 64, 65, 70, 71^. In support of Bcl-xL and Mcl-1’s critical role as gatekeepers against Bax/Bak activation, an elegant mouse genetic study found that while *Mcl-1 ^Bcl-2 +/–^* and *Bcl-xL ^+/–^Bcl-2 ^+/–^* mice are almost normal, *Mcl-1 ^+/–^Bcl-xL ^+/–^* mice displayed craniofacial defects ^72^.

Strikingly, Huang *et al*. found that Bad, the sensitizer protein, induced apoptosis as efficiently as the activator proteins tBid and Bim in the OctaKO derivative cells ^59^. Additionally, mutant activator BH3-only proteins with their BH3 domain replaced by that of Bad showed no difference from the wild-type activators in the induction of apoptosis in the OctaKO cells. Furthermore, tBid and Bim (both activators) as well as Bad (sensitizer) were found to induce apoptosis without distinguishing Bax from Bak, consistent with the conclusions of a recent *in vitro* study ^35^ but in contrast to an earlier study ^73^. Lastly, tBid and Bim are unable to accelerate the spontaneous activation of Bax in the Bcl-2 allKO cells, suggesting that there is no functional/physical interaction between the activator BH3-only proteins and Bax/Bak after the neutralization of the anti-apoptotic Bcl-2 proteins ^59^. These results are incompatible with the direct activation model and argue strongly that there is no distinction between direct activators and sensitizers and that the only target of the BH3-only proteins is the anti-apoptotic Bcl-2 proteins. These findings unify the functions of BH3-only proteins and provide an elegantly simple model for apoptotic signaling ( Figure 4B).

Of note, loss-of-function studies in mice have clearly demonstrated differential requirements for various BH3-only proteins *in vivo* ^74– 84^. These observations are consistent with the notion that the BH3-only proteins’ roles are determined by their expression level, location, various regulations, and affinity with different anti-apoptotic Bcl-2 proteins, etc.

## A bigger role for MOM in Bax and Bak activation

MOM has traditionally been viewed as a passive victim of activated Bax/Bak. However, some *in vitro* studies have suggested that MOM can serve as a platform for multiple interactions among the Bcl-2 family proteins, including tBid–Bax, tBid–Bcl-xL interactions, etc. ^31, 85, 86^. Results from the new series of studies, especially the finding that the TD (helix 9) of Bax/Bak was required and sufficient for Bax/Bak activation in the absence of all BH3-only and anti-apoptotic Bcl-2 proteins, suggested a much bigger role for the MOM in the regulation of Bax/Bak: the direct activator ^59, 65, 66^. In other words, the MOM itself functions as the instigator of Bax/Bak’s attack by directly recruiting and activating Bax/Bak. These results also suggested a much bigger role for helix 9: the trigger site. Importantly, the interaction between helix 9 and the MOM is spontaneous, stable, and easily detectable in the cell. On the other hand, the transient activator BH3-only–Bax/Bak interaction observed *in vitro* has been shown to be non-essential in cellular assays.

How does the MOM activate Bax? First, as Bax has been shown to exist in a dynamic equilibrium between the cytosol and mitochondria, and that Bcl-xL constantly retro-translocates Bax/Bak into the cytoplasm ^67, 68, 87^, it is essential that the anti-apoptotic Bcl-2 proteins are inactivated by the BH3-only proteins or other inhibitors before the MOM becomes a direct activator ^59, 66^. Second, a systematic mapping of the helices of Bax for their capacity to associate with the MOM indicated that helices 4, 5, 6, and 9 contain mitochondrial targeting signals (MTS) ^88^. It is not difficult to imagine that the MOM has the capacity to reconfigure the whole molecule through these multiple lipid–protein interactions to promote Bax/Bak homo-oligomerization upon the initial contact with helix 9. Third, as helix 9 of Bax was recently shown to form dimers in the MOM, such dimerization through helix 9 should provide further impetus for homo-oligomerization and activation of Bax/Bak molecules ^52, 89– 91^.

Thus, lipids are most likely the direct activator of Bax/Bak. Consistent with this suggestion, cardiolipin was found to be required for Bax activation ^29, 92^. Interestingly, it has been shown that non-ionic detergents, which somewhat mimic the lipid bilayer environment, are excellent activators of Bax/Bak ^12, 93^. It is therefore of great interest to test if certain lipid compositions can support Bax activation without the activator BH3-only proteins, even though the commonly used preparations prohibit such activity. A liposome composition that supports the constitutive activation of Bax in the absence of tBid should recapitulate what is observed in the cell.

MOM proteins may help recruit Bax to the MOM. VDAC2, despite earlier observations that it sequesters and suppresses Bak ^94, 95^, has recently been shown to be required for the mitochondrial translocation of Bax ^96, 97^. In addition, the mitochondrial fission regulator Drp1 has been shown to play a positive role in the regulation of Bax/Bak activation ^98– 100^. As both VDAC2 and Drp1 are present in the Bcl-2 allKO cells, it is possible that these proteins play a positive role in recruiting Bax/Bak along with the lipids to allow spontaneous localization ^59, 66^.

## *De novo* activation versus auto-activation of Bax/Bak

Based on results from a liposome study with an extended BH3 peptide and Bax, it has been hypothesized that active Bax uses its BH3 domain to directly bind and activate an inactive Bax to accelerate Bax activation. This putative feed-forward mechanism is termed “auto-activation” ^101^. In support of this hypothesis, during apoptosis, Bax/Bak undergo conformational changes and expose the BH3 domain ^17, 40^. Auto-activation has recently been suggested to be a critical feed-forward mechanism for Bax/Bak activation in response to DNA damage and other death stimuli ^36, 102^. However, the origin of the first active Bax/Bak molecule and the physiological significance of auto-activation are unclear. Furthermore, there are no experimental data to support the hypothesis that active Bax can recruit cytosolic Bax to the mitochondria.

Both the direct activation and MMS model probe the mechanism of Bax/Bak’s *de novo* activation. Although there is potential involvement of auto-activation as a feed-forward mechanism to sustain Bax/Bak activation through homo-oligomerization, it is not the triggering mechanism that initiates the activation of Bax/Bak *per se*. For example, although the “interconnected” model contains an element of auto-activation, the origin of the first active Bax/Bak molecules was attributed to direct activation ^36^.

## How do the BH3 mimetics work?

Thanks to a close collaboration between basic researchers and the pharmaceutical industry, numerous small molecules that mimic the structures of the BH3 domains of the BH3-only proteins have been developed to kill cancer cells and overcome resistance ^103^. For example, through NMR-based fragment screen and structure-guided design, ABT-737 was developed to mimic BadBH3, and it is highly selective against Bcl-xL, Bcl-2, and Bcl-w ^104^. Using similar strategies, Kotschy *et al*. designed S63845, a highly selective inhibitor of Mcl-1 ^105^. Remarkably, venetoclax, a BH3 mimetic and the first small-molecule drug that disrupts a protein–protein interaction, was FDA approved for the treatment of a subtype of chronic lymphocytic lymphoma ^103, 106– 109^. However, the obvious question is this: which interaction does it disrupt in the cell? According to the direct activation model, the Bcl-2 mimetic disrupts two interactions: activator BH3-only–Bcl-2 and Bax/Bak–Bcl-2. The liberated activator BH3-only proteins then directly activate the liberated Bax or Bak ^106^. However, this scenario is difficult to prove experimentally and is not consistent with the observation that combinations of BH3 mimetics against Bcl-xL and Mcl-1 are sufficient to kill OctaKO HCT116 cells ^59, 110^. Such an observation is explained by the MMS model, in which the BH3 mimetics neutralize Bcl-xL/Mcl-1 and allow the MOM to directly activate Bax/Bak. It is of interest, however, that over-expression of Bcl-xL confers resistance to the apoptotic effects of Bcl-xL-targeting BH3 mimetics, suggesting limitations of these small-molecule inhibitors ^111, 112^.

## How do the anti-apoptotic Bcl-2 suppress Bax/Bak activation?

It is well established that Bax exists mostly as an inactive and monomeric protein in cytosol ^14^. However, the spontaneous activation of Bax in the Bcl-2 allKO strongly suggests that Bax is constantly trying to land on the mitochondria through its C-terminal tail (α9) ^59, 66^, consistent with the observation of a natural tendency of Bax to move to the mitochondria ^67, 68^. It is also clear that Bcl-xL and Mcl-1 play a critical role in keeping Bax in the cytosol. How do Bcl-xL and Mcl-1 achieve this feat? Edlich *et al*. demonstrated that Bcl-xL has the ability to push the mitochondrially localized Bax molecules into the cytosol ^67^. Interestingly, Todt *et al*. demonstrated that Bak can also be retro-translocated by Bcl-xL, albeit at a much slower rate ^87^. However, the molecular mechanisms of such a retro-translocation activity remain unclear. Nevertheless, helix 9 of Bcl-xL may play a role in mediating this mysterious activity ^113^, through which the Bax molecule may be re-packed into a globular shape so that helix 9 can fit into the hydrophobic groove. Once this re-packing is finished, Bax is expected to leave the mitochondria and become cytosolic. The molecular details of such extraordinary activity will be a focus of future studies.

## The membrane (lipids)-mediated spontaneous model versus indirect and direct activation models

The MMS model combines some important elements from both the direct and the indirect activation models yet for the first time introduced a new interaction as the trigger for Bax/Bak activation ( Figure 2 and Figure 3, Table 1). First, in agreement with indirect activation, the MMS model proposes that there is no hierarchy among the BH3-only proteins and the only target of the BH3-only proteins are the anti-apoptotic Bcl-2 proteins. Second, similar to the direct activation model, the MMS model proposes that a *de novo* direct activation, instead of de-repression, is the mechanism of Bax/Bak activation. However, the MOM, instead of the activator BH3-only proteins, is the direct activator. Third, the MMS model proposes that helix 9 is the trigger site for Bax/Bak activation. Instead of a transient, “hit-and-run” interaction as proposed in the direct activation model, the interaction between Bax/Bak and the MOM is visible and stable and consists of multiple interfaces once homo-oligomerization initiates.

**Table 1.** Comparison of the three models of BH3-only-mediated Bax/Bak activation.

Model of Bax/Bak Activation	Targets of the BH3-only proteins	BH3-only– Bax/Bak Interaction	Activation Mechanism	Direct Activator	Trigger Site on Bax/Bak	Driving Force
**Indirect Activation** **(Displacement)**	Anti-apoptotic Bcl- 2 proteins	None	Displacement and De- repression	None	None	Stable BH3- only–Bcl-xL/Mcl-1 interaction
**Direct Activation**	Bax/Bak and anti- apoptotic Bcl-2 proteins	Yes	*De novo* and direct activation	Activator BH3- only proteins	Helices 1/6 or the hydrophobic groove	Transient BH3 (BH3-only)–groove (Bax/Bak) interaction
**Membrane** **(lipids)-mediated** **Spontaneous** **Activation**	Anti-apoptotic Bcl- 2 proteins	None	*De novo* and direct activation	Mitochondrial outer membrane (lipids)	C-terminal tail (helix 9)	Stable BH3- only–Bcl-xL/Mcl-1 interaction, free diffusion, and stable Bax/Bak α9-membrane interaction

Some questions remain to be answered: what are the molecular details of MOM-mediated spontaneous Bax/Bak activation? What are the dynamics of the helix 9–hydrophobic groove interaction in the cytosol? Can *in vitro* liposome assays recapitulate the MMS model? What are the mechanisms of retro-translocation?

## Conclusion

The balance among the Bcl-2 family members, namely the relative amounts of the BH3-only proteins and the anti-apoptotic Bcl-2 proteins, determines the fate of the cell through the activation of Bax and Bak ^59, 66^. According to the MMS model, during apoptotic signaling, once all the Bcl-xL/Mcl-1 molecules are neutralized by the BH3-only proteins, Bax/Bak spontaneously (through free diffusion) associate with the MOM, which acts as a direct activator to trigger Bax/Bak’s self-aggregation and pore formation ^59, 66^. This remarkably efficient and simple mechanism might provide new thinking on pharmacological intervention. For example, is it possible to manipulate the lipid composition or other properties of the MOM to either accelerate or slow down the attack by the Bax/Bak molecules? Also, since helix 9’s exposure and access to the MOM is essential to this process, is it possible to design small molecules to promote or suppress the dislodging of helix 9 from the hydrophobic groove of Bax/Bak, therefore modulating the targeting of these molecules to the mitochondria ^114, 115^?
